# Cloning, expression and functional characterization of prepared bovine, salmon, and cod basic fibroblast growth factor-2

**DOI:** 10.1038/s41538-025-00609-2

**Published:** 2025-11-21

**Authors:** S. Skrivergaard, M. E. Pedersen, A. Holck, A. Fagerlund, L. Axelsson, N. T. Solberg, M. K. Rasmussen, J. F. Young, M. Therkildsen, S. B. Rønning

**Affiliations:** 1 Department of Food Science, Aarhus University, Aarhus, Denmark; 2 Nofima AS, Raw Materials and Optimization, Ås, Norway; 3 Nofima AS, Food Safety and Quality, Ås, Norway

**Keywords:** Cell delivery, Expression systems

## Abstract

Growth factors (GFs) contribute to over 90% of the total expenses of medium costs. In this study, a codon-optimized gene encoding bovine, salmon, and cod FGF-2 was cloned into an *E. coli* expression vector. The bioactivity of the recombinant FGF-2 was tested in serum-free medium, demonstrating its ability to support MuSC proliferation and signaling pathways, ERK, and p38. Using serum-free medium with fetuin and Insulin–Transferrin–Selenium (ITS), all three in-house-produced recombinant FGF-2 variants showed positive effects on MuSC proliferation. Notably, bovine FGF-2 performed better using low concentrations (2 ng/mL) than the salmon and cod FGF-2, which required higher concentrations (17.5–70 ng/mL) to achieve the same effect on the cells. A synergistic relationship between bovine FGF-2 and the p38 inhibitor was revealed. These findings suggest that the medium cost from FGF-2 can be significantly reduced with optimized production and application strategies, supporting the large-scale viability of CM production.

## Introduction

The cell culture medium is a major limitation and cost driver for cultured meat (CM) development. Innovative, cost-efficient, and sustainable culture media are needed to improve our understanding of how medium components affect the final product. The medium cost remains a critical factor for CM viability^1,2^. An optimal growth medium should be affordable, available in large quantities, and support high cell performance. Various animal cell types can be used for CM, but their growth rates, metabolism, myogenic capacity, and genomic stability vary^3^. Primary skeletal muscle satellite cells (SCs) are the most promising for CM due to their ability to self-renew, expand, and differentiate^4^. These cells can be isolated in the lab and grown ex vivo while still retaining critical myogenic features^5^. CM production relies on SCs’ ability to regenerate and maintain muscle stemness, which can be compromised by suboptimal culture media. A basal formulation keeps cells alive short-term, but long-term proliferation without losing myogenic potential is challenging. Culture medium development has mainly been performed in and for the pharmaceutical industry, e.g., for therapeutic protein production, with other market requirements where the medium constitutes a small part of product costs. Developing a cell culture medium with the requirements of CM in mind is essential to accelerate the development of large-scale CM production. The medium is one of the most important factors underlying the near-term success of the CM industry. It is divided into two groups: (1) the basal medium, which is a buffered solution that contains glucose, amino acids, inorganic salts, and water-soluble vitamins, ingredients which can be generated by bacterial fermentation and obtained from plant sources and (2) specialized ingredients, such as growth factors, antioxidants, lipids, and hormones, that permit the long-term maintenance, proliferation, or differentiation of cells. In addition, traditional cell culture medium for cultivated meat application contains fetal bovine serum (FBS). This FBS is harvested from bovine fetuses at the slaughterhouse and contains growth factors, enzymes, lipids, hormones, carbohydrates, and proteins crucial for growth and cell differentiation. However, FBS supplementation is a limiting factor for the sustainable large-scale production of CM for human consumption. Serum is very expensive, and it raises ethical and safety concerns; the cost can be up to 96% of the total cost of cell growth medium. Supply is often lower than demand, and its composite and undefined nature can induce variability in cell growth. As such, cell cultivations should preferably be performed without the use of FBS^6^. The cell culture medium composition influences the growth of the cell, and the types of growth factors and hormones present in the cell culture medium critically maintain proliferation and differentiation. The absence of specific growth factors and hormones can make SCs lose their stemness^7^. Extensive research in the last decade has focused on reducing and replacing FBS with a chemically defined medium (serum-free medium, SFM) as part of good cell culture practice (GCCP)^8–10^. One commonly used serum replacement is Ultroser G. This is a semi-chemically defined serum replacement containing various growth factors, including fibroblast growth factors (FGFs), epithelial growth factor (EGF), and insulin-like growth factor (IGF). Previous results demonstrated that culturing SCs in Ultroser G extended the life span of viable cells^11^ and increased the number of SCs in primary cell cultures^12^. In previous work from our lab, the primary SCs were expanded to a cell culture medium with low glucose and only 2% FBS by adding Ultroser G as an FBS-replacement^13^. However, most commercially available serum replacements show lower performance and are only suitable for a limited number of cell lines, and they may even undesirably alter the cell phenotype^3,10^. Furthermore, most commercially available SFMs (including Ultroser G) and serum substitute alternatives adapted to MuSCs are not chemically defined, not food grade, and still present challenges in terms of cost and performance^9,14^. Recent research efforts aim to develop SFM using recombinant proteins, resulting in several different SFM formulations allowing high cell growth and differentiation capacity^15^. Although only low concentrations of growth factors are needed in the serum, they are rather costly, and as such, reducing their cost is critical. Key drivers for high costs are FGF-2 and transforming growth factor -β1 (TGF-β1)^16^. One approach to avoid the high serum costs is to adapt cells to fewer growth factors or to produce the recombinant growth factors via genetic modifications^17^ or through fermentation by genetically engineered microorganisms^16,18^. FGF-2 does not require post-translational modifications produced by eukaryotes for activity and can be produced in the bacterial host *Escherichia coli*, a widely used, inexpensive, and versatile recombinant expression host. FGFs are secretory, self-dimerizing heparin-binding growth factors that are associated with high-affinity tyrosine kinase receptors and low-affinity co-receptors; the heparan sulfate proteoglycans (HSPGs) located in the extracellular matrix, such as syndecan-4. Syndecan-4 and the cell surface receptor Integrin-β1 are essential for optimal FGF signalling^19^. FGFs are essential for SC function, and FGF-1, FGF-2, FGF-4, and FGF-6 can stimulate the expansion of cultured SCs^19,20^. FGF binding to FGFR activates the receptor and initiates signaling cascades^19^. Several signaling pathways are activated upon FGF binding, including p38, ERK, PI3 kinase, and Akt^19^. The p38 signaling pathway is necessary to activate the SCs, induce expression of myoblast determination protein 1 (MyoD), make the cells enter the cell cycle, and induce terminal differentiation^21^. Activation of ERK is necessary to allow the proliferation of SCs^19^. Studies where recombinant FGF have been used in medium for bovine SCs are still limited, although a study conducted in 2022 demonstrated the importance of FGF-2 compared to other growth factors on bovine SC proliferation^16^.

In this paper, we aim to produce recombinant bovine FGF-2 using *E. coli* suitable for bovine SC proliferation. We also cloned salmon and cod FGF-2 to investigate the impact of species specificity. Furthermore, we explored the involvement of p38 and ERK signaling pathways in the SC cell cycle. Finally, we explored the prospect of using the recombinant GF in an SFM.

## Results

### Expression and purification of FGF-2

**Fig. 1:**
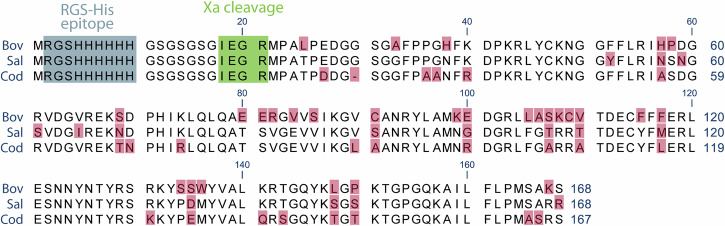
Amino acid sequence of the recombinant FGF-2 proteins produced in *E. coli.* Differences in amino acid sequence are marked in pink, the RGS-His epitope is marked in blue, and the Xa protease recognition site in green.

FGF-2, encoded by the *FGF2* gene, exists in several different isoforms resulting from alternative translation initiation sites. The high molecular weight variants are predominantly located in the nucleus, while the low molecular weight variant is the cytoplasmic and secreted variant^22^. A codon-optimized gene encoding the native low molecular weight 146 aa variant of bovine FGF-2 was cloned into an *E. coli* expression vector with a 22 aa N-terminal extension containing an RGS-His epitope (RGSHHHHHH) and a Factor Xa cleavage site (IEGR) (Fig. 1). In addition to the bovine FGF-2, the corresponding salmon and cod FGF-2 isoforms were produced. The mature salmon and cod proteins were 77% and 72% identical in amino acid sequence to the bovine version (Fig. 1).

While bacterial lysis under denaturing conditions (8 M urea) showed that recombinant proteins were expressed, initial attempts to generate lysates using bead beating in PBS resulted in low recombinant protein recovery due to the formation of inclusion bodies. However, lysis under native conditions using one freeze-thaw cycle followed by sonication resulted in a soluble target protein. No difference in expression was observed upon induction of expression with 0.2 mM and 1 mM IPTG and between bacterial culture growth at 30 °C and 37 °C. The yield was not affected by the addition of lysozyme (1 mg/mL), additional NaCl (1 M), Triton X-100 (0.25%), or glycine (50 mM) (data not shown).

**Fig. 2:**
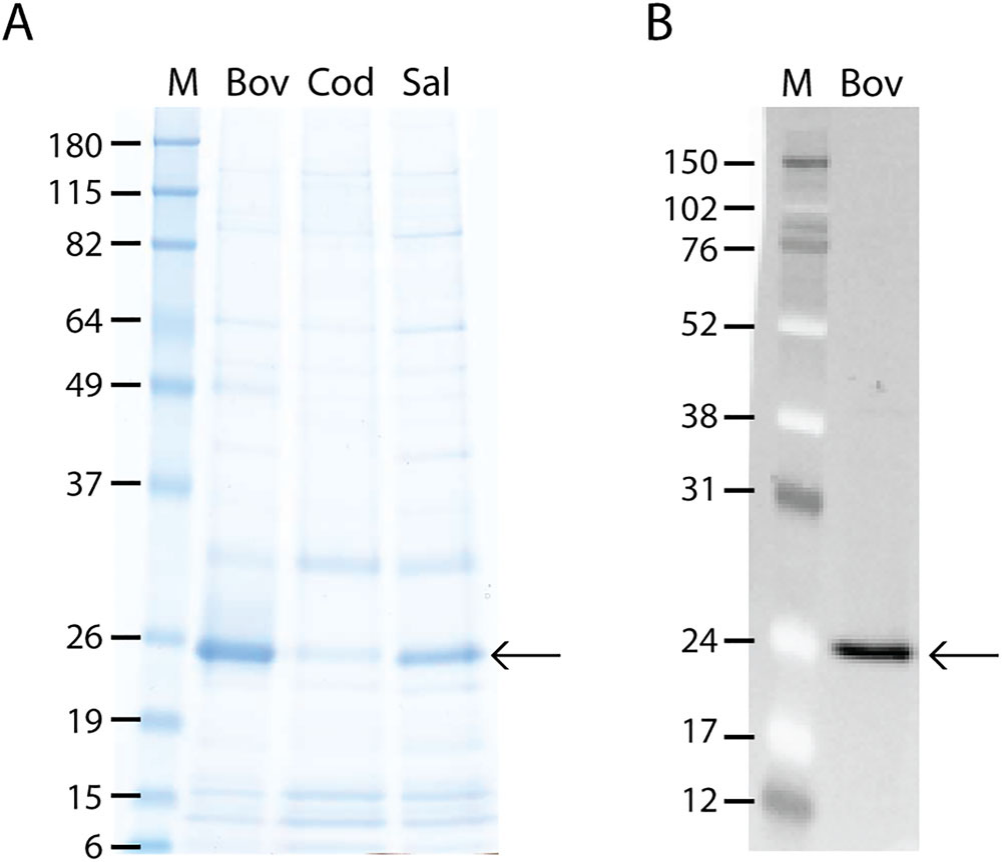
Protein expression of recombinant proteins. **A** Purified recombinant bovine (Bov), cod (Cod), and salmon (Sal) FGF-2, run on a SimplyBlue-stained SDS-PAGE gel. **B** α-RGB-His Western immunoblot of lysate containing recombinant bovine FGF-2. M; Protein size marker, with sizes shown in kDa. Arrows indicate the FGF-2 protein at 24 kDa.

The proteins were purified from *E. coli* cell lysates in a single-step purification enabled by the N-terminal His-tag, using Ni-NTA spin columns. The proteins were desalted using gel filtration and then lyophilized for stability during storage. Representative examples of SDS-PAGE gels showing proteins before and after Ni-NTA purification are presented in (Supplementary Fig. S1). Total yield after purification was 5–8 µg/mL for bovine FGF-2; however, a much lower yield was obtained for cod (2 µg/mL for cod FGF-2) and salmon (3 µg/mL for salmon FGF-2). Also, the purified recombinant proteins from bovine, cod, and salmon were 62%, 13%, and 34% pure (evaluated based on total protein bands) (Fig. 2), corresponding to 3 µg/mL, 0.3 µg/mL, and 1 µg/mL purified proteins. The predicted molecular weight for the recombinant proteins is 19 kDa. However, when the total amount of protein was examined using SDS-PAGE and immunoblotting of lysate using antibodies against the RGB-His epitope, the his-tagged recombinant protein was visualized as a 24 kDa protein (Fig. 2). The apparent higher molecular weight observed on SDS-PAGE may be due to anomalous migration behavior of FGF-2 or partial dimerization.

### Stimulation with FGF-2 influences bovine satellite cell proliferation and ERK and p38 signaling

**Fig. 3:**
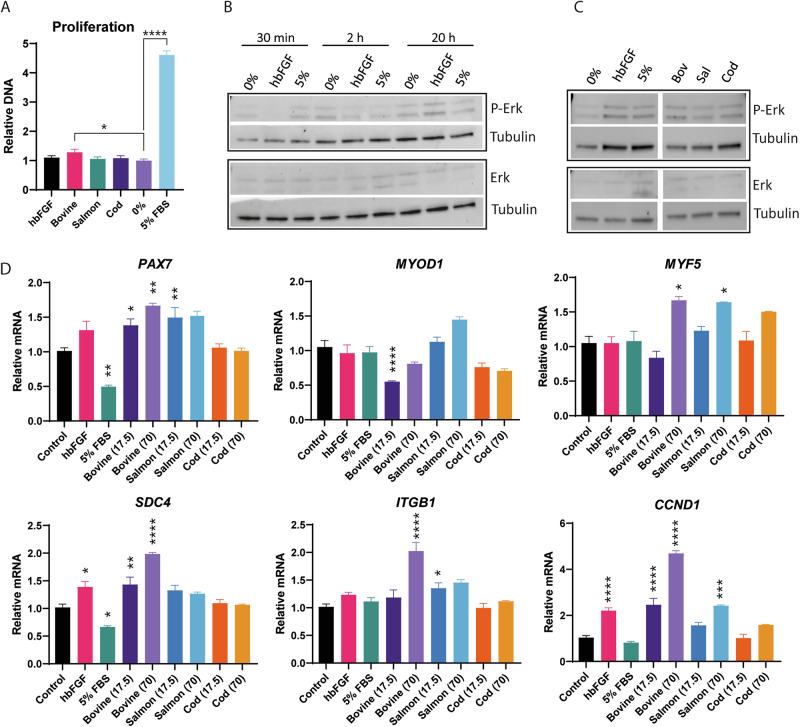
Effect of produced FGF-2 variants on proliferation, signaling pathways, and gene expression of myogenic markers and adhesion molecules. **A** The proliferation of cells incubated with different FGF-2 variants in basal media at 17.5 ng/mL for 72 h was measured using CyQuant DNA quantification. Cells were initially starved for 3 h in basal medium (0%) before the addition of the FGF-2 variants or 5% FBS. Relative DNA numbers are normalized against 0% control. Unpaired t-test was performed to test for significance compared with 0% basal medium, in which *p* < 0.05 (*), *p* < 0.01 (**), *p* < 0.001 (***), *p* < 0.0001 (****). All samples *n* = 8. Error bars are ±SEM. **B** Representative Western blots of cells incubated with 17.5 ng/mL hbFGF, 5% FBS, or basal medium alone (0%) for 30 min, 2, or 20 h. **C** Western blots of cells incubated with hbFGF or the three in-house FGF-2 variants at 17.5 ng/mL, 0% (basal medium) or 5% FBS for 20 h. Cells were initially starved for 3 h in basal medium (0%) before adding the FGF-2 variants or 5% FBS. Blots grouped in black boxes are from the same membrane, while re-arranged lanes that are non-adjacent are delineated by a white spacing. **D** qPCR of cells incubated with 17.5 ng/mL hbFGF, 5% FBS, or with the three in-house FGF-2 variants at 17.5 ng/mL and 70 ng/mL for 20 h in basal medium. Data is normalized against the basal medium control sample (0%). One-way ANOVA with Dunnett’s multiple comparisons compared with 0% control sample (or 0.0 ng/mL) in which *p* < 0.05 (*), *p* < 0.01 (**), *p* < 0.001 (***), *p* < 0.0001 (****). All samples *n* = 12, except 70 ng/mL samples *n* = 3. Error bars are ±SEM.

Initially, the three FGF-2 variants were screened alongside a commercial human FGF-2 variant (hbFGF) in different concentrations (4.4–70 ng/mL) in a serum-free basal medium by measuring DNA content after 48 h and 72 h of bovine satellite muscle cell proliferation (Supplementary Fig. S2). These initial data showed no significant increases in proliferation when compared to the basal medium, yet indicated a trend in growth stimulation in the 17.5–70 ng/mL concentration range. Additional testing with 17.5 ng/mL FGF-2 showed that the bovine variant could significantly increase the amount of DNA after 72 h when cells were serum-starved for 3 h before exposure to FGF-2 (Fig. 3A). This increase was, however, very small compared to the 5% FBS control.

The phosphorylation and activation of the ERK signaling pathway within the bovine SCs using 17.5 and 70 ng/mL of FGF-2, using immunoblotting, were then examined. Initially, exposure to hbFGF or 5% FBS for 30 min, 2 h, or 20 h was tested, and the highest phosphorylation of ERK was observed after 20 h (Fig. 3B), especially apparent with hbFGF (Supplementary Fig. S9). The three FGF-2 variants recombinantly produced in-house were also evaluated after 20 h at 17.5 ng/mL (Fig. 3C), all showing phosphorylation of ERK when similar to the commercial hbFGF. The three recombinant FGF-2 variants were also tested at 70 ng/mL, although there was no apparent change in ERK activation compared with 17.5 ng/mL (Supplementary Fig. S3). The bovine FGF-2 type showed the highest activation of the three when blots were quantified (Supplementary Fig. S9), although this was only a trend and not statistically significant.

To further investigate the molecular effect of the FGF-2 variants, the relative gene expression of three myogenic markers *PAX7*, *MYOD1*, and *MYF5*, the essential transmembrane receptors *SDC4* and *ITGB1*, and the proliferative marker *CCND1* was analysed after 20 h of incubation with the FGF-2 variants at 17.5 ng/mL and 70 ng/mL (Fig. 3D). Bovine and salmon FGF-2 induced a significant upregulation of *PAX7* expression, while 5% FBS decreased it compared to basal medium control. *MYF5* expression was also increased by bovine and salmon FGF-2, but only at 70 ng/mL, while *MYOD1* was downregulated only when 17.5 ng/mL bovine FGF-2 was used. The bovine FGF-2 significantly upregulated both *SDC4* and *CCND1* at 17.5 ng/mL, with an increased effect at 70 ng/mL, while also increasing *ITGB1* expression at this level. This is in contrast to the commercial hbFGF. A similar trend was notable with the cod FGF-2. Notably, 5% of FBS decreased *SDC4* expression without impact on *CCND1*, while the bovine variant at 17.5 ng/mL increased *SDC4* and *CCND1* to the same level as the commercial hbFGF. Altogether, we show that the bovine FGF-2 at 17.5 ng/mL slightly increased muscle cell proliferation and significantly activated the ERK signaling pathway. Additionally, bovine FGF-2 upregulated key myogenic markers and proliferative genes, indicating its potential in muscle development.

### ERK and p38 inhibitors indicate FGF-2 signaling

**Fig. 4:**
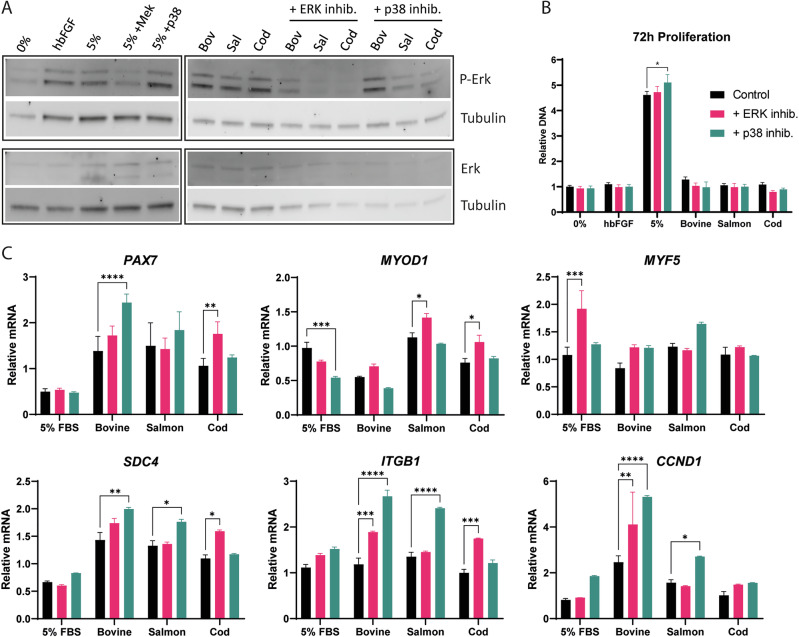
Effect of ERK and p38 inhibitors alongside FGF-2 incubation. **A** Western immunoblots of cells incubated with different FGF-2 variants at 17.5 ng/mL, 5% FBS, or basal medium alone (0%) for 20 h, with the addition of ERK and p38 inhibitors as indicated. Blots grouped in black boxes are from the same membrane**. B** CyQuant DNA quantification of cells incubated with different FGF-2 variants at 17.5 ng/mL, 0% or 5% FBS with or without ERK and p38 inhibitors for 72 h. Relative DNA numbers are normalized against 0% control. Control samples *n* = 8, while inhibitor samples *n* = 4. **C** qPCR of cells incubated with the three in-house FGF-2 variants at 17.5 ng/mL or 5% FBS with or without ERK and p38 inhibitors for 20 h in basal medium. Data is normalized against the basal medium control sample (0%). Cells were initially starved for 3 h in basal medium (0%) before adding the FGF-2 variants or 5% FBS, while inhibitors were added 2 h into this starvation. Two-way ANOVA with Dunnett’s multiple comparisons relative to the control sample without inhibitor for each treatment group, in which *p* < 0.05 (*), *p* < 0.01 (**), *p* < 0.001 (***), *p* < 0.0001 (****). All qPCR samples *n* = 12, except inhibitor samples *n* = 3. Error bars are ±SEM.

The ERK and the p38 pathways are two of the significant canonical signaling cascades activated by FGF-2. To elucidate their importance in the observed FGF-2-mediated gene upregulation, the effect of the produced FGF-2 variants was tested when cells were also treated with either an ERK or p38 inhibitor simultaneously. After establishing that the recommended inhibitor concentration range did not have apparent cytotoxic effects (Supplementary Fig. S4), the phosphorylation of ERK was analyzed using immunoblotting with the ERK and p38 inhibitors added at 1 nM and 100 nM, respectively (Fig. 4A and Supplementary Fig. S5). Here, the ERK inhibitor was observed to effectively inhibit the phosphorylation of ERK mediated by the three in-house FGF-2 variants and 5% FBS (Supplementary Fig. S9). The p38 inhibitor also decreased the phosphorylation of ERK in cells treated with the three FGF-2 types, but not in cells treated with 5% FBS, in which phosphorylation was highly increased. Furthermore, p38 inhibition also increased satellite cell proliferation in 5% FBS, while neither the ERK nor p38 inhibitors showed any effects after 72 h in any of the other cell growth conditions (Fig. 4B).

Surprisingly, neither inhibition of ERK nor p38 abrogated the upregulation of *PAX7*, *SDC4*, *ITGB1*, and *CCND1* induced by the bovine FGF-2 variant (Fig. 4C). Both inhibitors further increased the expression of *ITGB1* and *CCND1* induced by the bovine FGF-2, while p38 inhibition also increased *PAX7* and *SDC4* expression. Similarly, the p38 inhibitor also increased *SDC4, ITGB1*, and CCND1 expression in salmon FGF-2 samples, while ERK inhibition did so for *PAX7, MYOD1, SDC4*, and *ITGB1* in the cod FGF-2-treated cells. SCs incubated with 5% FBS seemed to react very differently to this, with downregulation of *MYOD1* when exposed to the p38 inhibitor, while ERK inhibition increased *MYF5* expression. Altogether, we show that inhibiting the ERK and p38 pathways did not completely block ERK phosphorylation or gene upregulation induced by FGF-2 variants. Interestingly, p38 inhibition increased the expression of several myogenic markers and proliferative genes.

### In-house-produced FGF-2 effectively increases proliferation in SFM

**Fig. 5:**
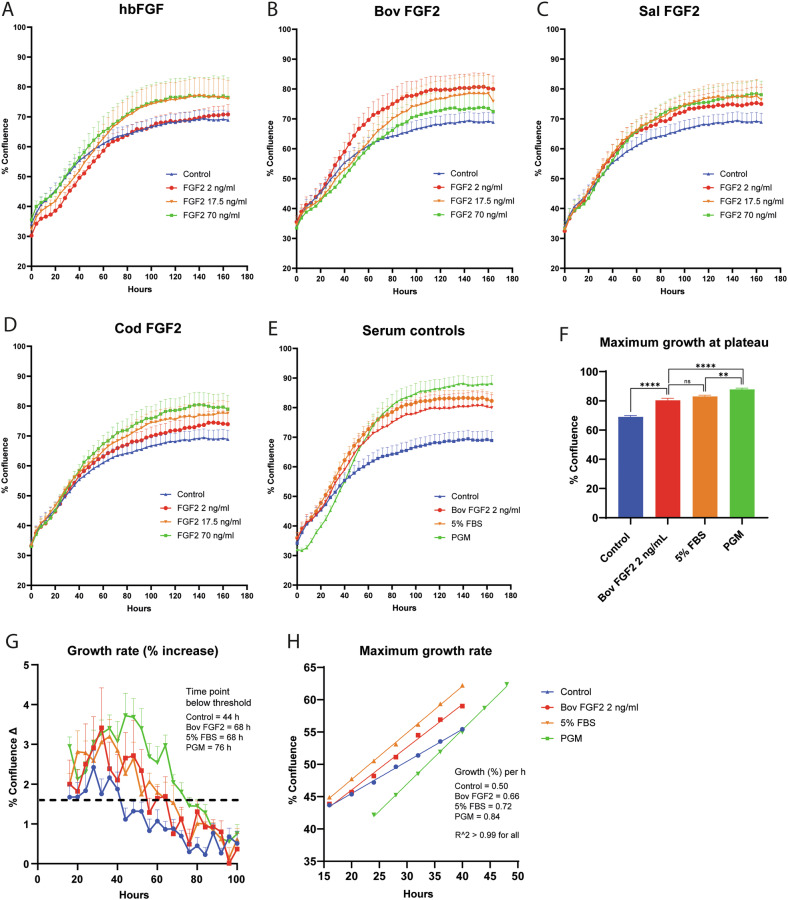
IncuCyte® live-cell proliferation assays using FGF-2 in combination with fetuin and ITS. **A**–**D** FGF-2 variants were added at 2 ng/mL, 17.5 ng/mL, or 70 ng/mL concentrations or left out (control) in basal SFM with fetuin (600 µg/mL) and ITS (1×). **E** The best-performing sample, Bovine FGF2 at 2 ng/mL, was compared with serum-containing medium types 5% FBS and PGM. **F** The maximum confluence values achieved from the samples (**E**) were compared (combined data from the plateau at time intervals 132–164 h). **G** The growth rate (% increase between 4 h time points, Δ) was graphed for the samples in **E**. Threshold in (**G**) is set at 1.6%, which is the mean standard deviation value of Bovine FGF2 samples in the high-variation interval 16–64 h. **H** Maximum growth rate in the interval 16–40 h was determined using the mean numbers from (**E**) by calculating the slope value (% increase per h) of a linear regression model. Data (% confluence) per time-point is: all FGF-2 samples *n* = 6, control *n* = 14, 5% FBS *n* = 12, PGM *n* = 18. Error bars are ±SEM. One-way ANOVA with Tukey’s multiple comparisons in (**F**) was performed on pooled data from the time interval 132–164 h, in which *p* < 0.05 (*), *p* < 0.01 (**), *p* < 0.001 (***), *p* < 0.0001 (****).

As adding the in-house produced FGF-2 variants as singular components into basal medium alone did not induce significant cell proliferation, we aimed to test the FGF-2 applicability in a more relevant cell culture medium. Recently, we described an optimized simple SFM containing fetuin and Insulin–Transferrin–Selenium (ITS), along with FGF-2 at a low concentration of 2 ng/mL^23^. Therefore, the in-house FGF-2 variants and the commercial hbFGF were tested in 2 ng/mL, 17.5 ng/mL, and 70 ng/mL concentrations in a similar SFM. A multi-day label-free live assay measured the resulting bovine satellite cell proliferation (Fig. 5A–D). All tested FGF-2 variants enhanced cell proliferation when added to the basal SFM with ITS (1×) and fetuin (600 µg/mL), compared to controls without FGF-2. However, the effectiveness varied among the different FGF-2 variants. For the cod FGF-2, there was a gradual increase in proliferation with increasing FGF-2 concentrations, while for hbFGF and salmon FGF-2, similar effects were observed at 17.5 ng/mL and 70 ng/mL. Interestingly, the opposite was observed for the bovine FGF-2, showing an inverse relationship, with the highest proliferation at the lowest concentration of 2 ng/mL (Fig. 5B). In fact, at the lowest concentration, the bovine FGF-2 displayed the highest stimulation of cell proliferation of all the FGF-2 variants. The bovine FGF-2 at 2 ng/mL was then compared against two serum-containing medium, 5% FBS and PGM (2% FBS and 2% Ultroser G), in which it showed comparable performance with 5% FBS (Fig. 5E). Indeed, when the highest confluence numbers achieved at the plateau (132–164 h) were compared, it was evident that bovine FGF-2 significantly increased proliferation above control while there was no difference between the FGF-2 and 5% FBS (Fig. 5F). The growth medium with 2% Ultroser and 2% FBS performed the best with significantly higher confluence numbers than the FGF-2 and 5% FBS. This medium also sustained proliferation for a longer time before plateauing (after ~76 h) (Fig. 5G) while also achieving the highest maximum growth rate (0.84% per hour) (Fig. 5H). The growth data for the bovine FGF-2 and 5% FBS were quite comparable here, with similar sustained growth rates (above a 1.6% threshold), both plateauing after 68 h and maximum growth rates of 0.66% and 0.72% per hour, respectively. The kinetics for these two treatments were much better than those of the control medium, which plateaued after only 44 h and had a maximum growth rate of 0.50% per hour. Cells proliferating in both commercial and homemade FGF-2-containing media were similarly able to differentiate as cells grown in growth media when differentiation was initiated after 6 days (Supplementary Fig. S7).

## Discussion

In this study, we successfully designed, produced, and validated three different variants of FGF-2 growth factors with protein sequences originating from bovine, cod, and salmon. Using a simple version of a previously developed SFM with fetuin and ITS^23^, there was an apparent positive effect when all three in-house-produced recombinant FGF-2 variants were added. There was, however, a notable difference in their concentration optima, as the bovine FGF-2 had the highest increase in proliferation at the lowest concentration (2 ng/mL). At the same time, the salmon and cod FGF-2 worked better in the 17.5–70 ng/mL range. This is an important parameter, as the much lower concentration directly translates into significantly lower medium costs attributed to the FGF-2 addition. This also reflects that for the technology, it is important to take into account cross-species differences when saving costs, and only species-specific growth factors should be used for growth stimulation. Notably, the concentrations used were based on total yield protein concentrations. Still, the purity of the recombinant proteins differed among the species, with salmon and cod showing less yield of the 24 kDa protein. This could explain some of the differences observed, especially in the ERK activation and gene expression data, where only one concentration was used. However, the large concentration range of 2–70 ng/mL used in the IncuCyte® live assay still confers that the best performer was the bovine FGF-2, as FGF-2 values for cod and salmon at 70 ng/mL would still be much higher than bovine FGF-2 at 2 ng/mL. This infers that the bovine FGF-2 had a significant effect at an even lower concentration than 2 ng/mL. When the concentration was adjusted according to the band quantified using the SDS-PAGE, no difference in growth was shown for the highest concentrations of salmon and cod compared with the protein concentration measured, total yield concentration (Supplementary Fig. S6). In our previous study^23^, the SFM was also optimized using only 2 ng/mL in-house made FGF-2; however, in that study, the basal medium used was DMEM/F12, a formulation combining high glucose, amino acids, and vitamins, significantly improving performance. As low-glucose DMEM was used with the serum-free components (also without the bovine serum albumin) in this research, we suggest that the multi-day proliferation could be improved further using the DMEM/F12 with the albumin added as the basis for the SFM. The bovine FGF-2 did, however, still perform similarly to 5% FBS, showing ERK phosphorylation and upregulation of *PAX7, SDC4, ITGB1*, and *CCND1*. Syndecan-4 (*SDC4*) is a well-known regulator of skeletal muscle growth and tissue repair^24–27^. In SDC4 knock-out mice, the loss of MAPK signaling prevented cell activation and proliferation^28–30^. FGF-mediated ERK activation is potentiated by integrin-β1 and syndecan-4 in muscle SCs^19^. Syndecan-4 is essential for FGF signaling and proper satellite cell function^31^ and can directly modulate FGF-2 signalling^24^, perhaps through an increased clustering-dependent internalization^32^. Furthermore, syndecan-4 can regulate cell surface levels of FGFRs and integrins in endothelial cells and fibroblasts^33,34^. Integrin-β1 (*ITGB1*) is directly involved in proper FGF-2 signalling^35^ and has been observed to have a higher expression in both bull calf and dairy cow SCs when grown in SFM compared with 10% FBS-containing medium^36^. The original SFM also showed a high upregulation of the proliferative markers *MKI67, CCNA2, CDC20*, and *CCND1*, with increased expression of enriched mitotic cell cycle pathways compared to a basal medium control^23^. Interestingly, the gene expression data suggested that the FGF-2-mediated increase in *PAX7, SDC4, ITGB1*, and *CCND1* expression was enhanced by the p38 inhibitor. Additionally, the reduced expression of *MYOD1* in the FGF-2 (bovine) samples was further reduced with the p38 inhibitor, significantly reducing *MYOD1* in the 5% FBS samples. Furthermore, the p38 inhibitor seemed to induce a slightly higher ERK activation observed in the immunoblotting while significantly enhancing proliferation in 5% FBS-treated cells. The ERK inhibitor, however, did not show any noticeable effects other than an effective reduction in ERK phosphorylation, as expected. Surprisingly, it had no adverse effects on proliferation despite induced expression of *CCND1* and the *ITGB1* receptor, yet it did induce a slightly higher *MYOD1* expression in FGF-2-treated cells and higher *MYF5* expression in the 5% FBS treatment.

ERK activation through FGF-2 signaling in SCs is required for proliferation and G1 to S-phase transition, as it increases cyclin D1 and reduces p21, yet this is insufficient for SC expansion^19^. Likewise, activation of p38 is required for SC activation, MyoD induction, and cell cycle entry^19^. However, one canonical function of p38 in SCs is to induce myogenic differentiation partly by activating MEF2C^37,38^. Furthermore, elevated p38 signaling has been observed to result in reduced FGF responsiveness in SCs from aged mice, which promotes terminal differentiation and reduces self-renewal by increasing MYOD, while reducing the p38 activity permits asymmetric division and partially rescues self-renewal of the SCs^39,40^. It has also been shown that p38 has apoptotic effects in endothelial cells when the AKT pathway is blocked, while inhibiting p38 attenuated this effect^41^. In astroglial cells, the ERK and p38 pathways were observed to work independently and antagonistically. The FGF-2-induced process extension of the cells was greatly enhanced when p38 was inhibited^42^. These mechanisms might explain the observed cell behavior in the present study, in which blocking the p38 pathway could result in higher FGF-2-mediated ERK activation and downstream effects like higher *SDC4, ITGB1, CCND1* gene expression, and enhanced proliferation. Contrarily, blocking the ERK pathway might shift cell signaling towards the p38 pathway, with higher expression of *MYOD1* and *MYF5* necessary for the transition into terminal myogenic differentiation, yet without major effect on the proliferative capacity in the timeframe tested here. This might suggest additional FGF-2-mediated pathways other than ERK that confer cell proliferation, e.g., FGF-2 has been observed to activate TRPC (transient receptor potential canonical) and Ca^2+^ signaling, leading to SC activation^43^. However, the induction of myogenesis by inhibiting ERK has been quite robustly established in other studies^44,45^.

Some limitations of this study should be noted, such as the accuracy of FGF-2 concentration measurements and purity, as well as the quantitative analysis of Western blots due to the limited experimental size, which prevented a robust statistical analysis. Future work will optimize purification for higher purity and reproducibility; however, the current preparations, despite partial purity, demonstrated clear bioactivity in functional assays. Also, the FGF-2 used in this manuscript contained a 22 aa N-terminal extension containing an RGS-His epitope (RGSHHHHHH) and a Factor Xa cleavage site (IEGR), and whether this tag has affected the results is unknown. Removing this tag might further increase the cost if used for up-scale production. The low stability of FGF-2 in a culture medium at 37 °C is also a factor that could be considered^46^, as FGF-2 is prone to degradation and loss of bioactivity at physiological temperatures, which can lead to frequent media changes and increased costs. The addition of albumin or fetuin as a stabilizing protein in the activation assays might have altered the FGF-2 stability and response^8,23^. This could also explain why the proliferative effect of the FGF-2 types was only observed in the designed SFM and not in the basal medium. Additionally, mutated versions of FGF-2 have been designed to significantly enhance stability and performance^47^, which could further limit this issue. These additions with multiple time points over a more extended incubation period would likely improve the acquired insights into the effects of FGF-2 signaling and activation of the p38 and ERK pathways in long-term cell cultivation and should be considered in future studies. Recently, recombinant FGF-2 production was optimized using the *E. coli* BL21 (DE3) plysS strain and was tested in a large-scale 500 L bioreactor with high yield and purity^48^. Furthermore, a small lab-scale production of FGF-2, similar to the current study, was calculated to be in the 7 USD per mg purified protein range, which was several hundred-fold cheaper than commercial suppliers^16^. This might suggest that the medium cost contribution from FGF-2 addition alone is an issue that can be solved practically with simple, scalable production systems. The FGF-2 variants with species-specific sequences from cod, salmon, and bovine origins produced in this study could activate ERK and enhance the proliferation of bovine SCs in an SFM. The bovine version had the highest effect of the three in a low concentration of 2 ng/mL, with better performance than a commercial FGF-2. It could be one further step towards an inexpensive, chemically defined SFM for cultivating muscle cells for cultivated meat and cell research.

## Methods

### Cloning and purification of FGF-2

The gene encoding bovine (*Bos taurus*) basic FGF-2 (aa 10–155; Uniprot accession P03969), codon-optimized with the OptimumGene algorithm (GenScript, NJ, USA), was synthesized with an upstream sequence encoding the RGS-His epitope, a Factor Xa cleavage site, and an ATG codon (Supplementary Table S1), cloned into the *Nde*I/*Eco*RI restriction sites in the pET-30a(+) plasmid (Novogene Co, China) downstream of the T7 promoter, and verified by Sanger sequencing. The corresponding constructs were also prepared for the salmon (*Salmo salar*) and cod (*Gadus morhua*) basic FGF-2 (aa 10–155) variants (NCBI accessions XP_014067501 and XP_030223479). The manuscript refers hereafter to these constructs, such as Bov, Sal, and Cod.

The constructed plasmids were introduced into *E. coli* OneShot BL21(DE3) (Invitrogen, MA, USA), in which expression of the T7 RNA polymerase gene is mediated by the addition of IPTG. *E. coli* were grown, and FGF-2 protein was produced as described by Rassouli^18^ and Protocol 6 in The QiaExpressionist^49^. *E. coli* was grown in tryptic soy broth (TSB) with 2% glucose and 25 ppm kanamycin at 37 °C at 200 rpm to an OD_600_ = 0.3. FGF-2 protein production was induced by adding IPTG to 0.2 mM or 1 mM and growing the bacteria 4–5 h before harvesting by centrifugation at 4000×*g* for 20 min. Pellets, if not used immediately, were stored at −80 °C. Thawed pellets were resuspended in 50 mM NaH_2_PO_4_, pH 8, 300 mM NaCl, and 10 mM imidazole, frozen in ethanol at −80 °C, and then thawed in cold water, and sonicated (Microson Ultrasonic cell disruptor XL 2005) on ice for 6 × 10 s. The lysate was centrifuged at 10,000×*g* for 20 min at 4 °C, and the supernatant resulting from lysis of a pellet from 50 mL bacterial culture was applied to a Ni-NTA Spin column from the Ni-NTA Spin Kit (Qiagen, Germany), which has a capacity of up to 300 µg of 6xHis-tagged protein. The column was washed four times with buffer containing 50 mM imidazole, and the purified protein was eluted by increasing the imidazole concentration to 250 mM. The eluted protein was desalted by gel filtration (Zeba Spin Desalting Columns, Thermo Fisher Scientific, MA, USA) and dissolved in 50 mM Na_2_HPO_4_ buffer, pH 8. The eluate was aliquoted and freeze-dried (Martin Christ Gefriertrockningsanlagen GmbH, Niedersachsen, Germany) after adding 0.125 mM β-mercaptoethanol and 0.025% Tween 20. Proteins from the corresponding constructs containing the salmon *FGF2* and cod *FGF2* genes were purified similarly.

### SDS polyacrylamide gel electrophoresis (PAGE) and Western immunoblotting

Samples were subjected to SDS-PAGE using the Invitrogen NuPAGE 12%, Bis-Tris, 1.0 mm, Mini Protein Gels system, essentially as described by the manufacturer (Invitrogen, MA, USA). Samples were mixed with Invitrogen NuPAGE LDS Sample Buffer (4×) and NuPAGE Sample Reducing Agent (10×), heat-treated at 70 °C for 10 min, and subjected to electrophoresis at 200 V for 50 min and stained with SimplyBlue SafeStain (Thermo Fisher Scientific, MA, USA). Benchmark pre-stained protein standard (Thermo Fisher Scientific, MA, USA) was used for molecular mass determinations. The purity of proteins (%) was determined by quantification of the specific 24 kDa band as a percentage of all bands from a SimplyBlue-stained gel using the ImageQuantTL software (Cytiva, MA, USA). Protein concentrations of the total yield of recombinant FGF-2 were determined using the Bio-Rad DC Protein Assay (Bio-Rad, CA, USA), and these numbers were used for the SC culture experiments. To confirm the presence of the correct proteins, some gels were immunoblotted using iBlot Transfer Stack, nitrocellulose (Invitrogen, MA, USA). The primary antibody was directed against the RGS-His epitope (Qiagen cat no 34650, Hilden, Germany), and the secondary antibody used was ECL Plex Goat anti-Mouse IgG Cy3, Cytiva (Danaher Corporation, DC, USA).

### Primary skeletal muscle satellite cell isolation and cell culture

Bovine SCs, used to screen for bioactivity, were isolated from beef sirloin (*Musculus longissimus*) provided by Nortura AS (Rudshøgda, Norway) using a published protocol^50,51^. In summary, a muscle biopsy weighing approximately 1–2 g underwent a series of enzymatic digestions. Initially, it was treated with 0.72 mg/mL collagenase in low-glucose DMEM (Dulbecco’s Modified Eagle Medium) containing GlutaMAX (Thermo Fisher Scientific, MA, USA) along with 10,000 units/mL penicillin/streptomycin (P/S) and 250 μg/mL amphotericin B. This digestion occurred for 1 h at 37 °C with gentle shaking at 70 rpm. Subsequently, the tissue underwent a 25-min digestion with 0.05% trypsin/EDTA. After each digestion step, 10% FBS was added to inactivate the enzymes, and this process was repeated three times. The resulting cell pellets were combined and resuspended in 3 mL low-glucose DMEM supplemented with 10% FBS, 250 μg/mL fungizone, and 10,000 units/mL P/S. To purify the SCs while removing fibroblasts, the cells were seeded onto uncoated 25 cm² culture flasks for 1 h at 37 °C. Fibroblasts adhered to the plastic surface, allowing the non-adherent SCs to be collected. These SCs were transferred to cell flasks coated with 1 mg/mL Entactin–Collagen–Laminin (ECL; Merck Millipore MA, USA). Upon reaching approximately 80% confluence, the cells were harvested and stored in liquid nitrogen with a freezing medium (8% dimethyl sulfoxide in DMEM) until further use. After thawing, the SCs were conditioned, maintained, and seeded (all experiments) in proliferative growth medium (PGM) containing low-glucose DMEM supplemented with 2% FBS, 2% Ultroser G, 250 μg/mL Fungizone, and 10,000 units/mL P/S.

Initial cell screening experiments were conducted on proliferating cells for 48 h and 72 h. The cells were seeded in ECL-coated 96-well microplates (3000 cells/well) in 100 µL/well PGM. After an overnight attachment, the cells were washed with PBS before the FGF-2 (4.4–70 ng/mL) was added to serum-free basal medium (low glucose DMEM), only with antibiotics. Gibco human heat-stable bFGF recombinant protein (hbFGF; Catalogue No. PHG0367, Fisher Scientific, NH, USA) was used as a positive control. Following the manufacturer's protocol, cell proliferation (DNA content) was measured using the CyQuant cell proliferation assay (Invitrogen, MA, USA). Fluorescent signals were measured using a Synergy H1M microplate reader (BioTek Instruments, VE, USA/Agilent Technologies, CA, USA).

The final long-term proliferation experiment used the IncuCyte® S3 Live-Cell Analysis System (Sartorius AG, Göttingen, Germany) for real-time confluence monitoring. The cells were seeded at 1000 cells/well on an ECL-coated 96-well plate. The medium was removed 24 h after seeding, and the attached cells were washed with PBS before being treated in triplicate with the different FGF-2 growth factors in basal serum-free medium (DMEM) with fetuin (600 µg/mL) and ITS (1×). The cell confluence was then monitored using IncuCyte S3 (Sartorius AG, Göttingen, Germany), placed in an incubator at 37 °C and 5% CO_2_. Cells were differentiated in low-glucose DMEM containing GlutaMAX, 2% FBS, 25pmol Insulin, 10,000 units/mL P/S, and 250 μg/mL amphotericin B.

### Western blotting of cell cultures

SCs were seeded at a density of 100,000 cells/well in 6-well plates and allowed to attach overnight. Cells were initially serum-starved for 3 h in the basal medium before adding the FGF-2 variants or 5% FBS, while inhibitors were added 2 h into this starvation. Incubation times of 30 min, 2 h, or 20 h were tested. The cells were washed twice with PBS before lysis in 150 µL NP-40 buffer containing 4-(2-Aminoethyl) benzenesulfonyl fluoride hydrochloride (AEBSF) (1:100) and phosphatase cocktail inhibitor II (1:200, Sigma-Aldrich, MI, US). Protein concentration was determined using the DC Protein Assay (BioRad, CA, USA) using 96-well plates. Extracted proteins were used for Western blotting. Equal amounts of protein were loaded to each lane (~2 µg per lane) of SDS-PAGE NuPage 4–12% Bis-Tris gels (Thermo Fischer, MA, USA) using 3-(*N*-morpholino) propane sulfonic acid (MOPS) SDS running buffer (Invitrogen, NP0001, MA, USA) at 200 V for approximately 50 min. Following electrophoresis, the proteins were transferred onto nitrocellulose membranes using an iBlot Gel Transfer Device (Invitrogen, MA, USA). All membranes were blocked with a 2% ECL Advance blocking agent (GE Healthcare, IL, USA) in TBS-tween for 1 h at room temperature (RT). The primary and secondary antibodies were diluted in a 0.2% blocking agent and incubated for 1.5 h at RT (or overnight at 4 °C) with gentle shaking. Membranes were washed 3 × 10 min with TBS-tween after both incubations. ECL plex™ Rainbow™ Fluorescent marker from Cytiva (#RPN850E, MA, USA) was used as a molecular weight marker. Proteins were scanned and visualized using G: BOX Chemi XX6/XX9-(Syngene, India). Antibodies and concentrations used were: α-tubulin (1:10,000, #T5168, Sigma Aldrich, MI, USA), α-p44/42 (1:1000, #4695, Cell Signalling, MA, USA), α-p-p44/42 (1:1000, #9101, Cell Signalling, MA, USA). All immunoblots can be seen uncropped and unedited in the Supplementary Fig. 8. Band intensities were quantified by the densitometric Gel Analysis tool in ImageJ, in which the area under the curve was used. The values for the P-Erk and Erk were the sum of the two isoform bands.

### qPCR

Total RNA was extracted using the RNeasy MiniKit (Qiagen, #74104, Germany) following the manufacturer’s instructions. Initially, the culture medium was aspirated, and the cells were washed with PBS before being lysed with 350 mL of lysis buffer containing 2 M Dithiothreitol (DTT). According to the manufacturer’s protocol, cDNA was synthesized from 2 ng to 10 ng of total RNA using TaqMan Reverse Transcription Reagents (Invitrogen, MA, USA). qPCR analysis was then performed using the TaqMan Gene Expression Master Mix (Thermo Fisher Scientific, CA, USA) and the QuantStudio5 PCR System (Applied Biosystems, CA, USA). The amplification protocol included an initial step at 50 °C for 2 min, followed by denaturation at 95 °C for 10 min, and then 45 cycles of denaturation at 95 °C for 15 s, annealing, and amplification at 60 °C for 1 min. RT-PCR analyses were conducted on at least three replicates. ΔCt values were calculated according to MIQE guidelines, and relative gene expression (fold change) was determined using the 2^−ΔΔCt^ method^52,53^. Relative gene expressions were normalized to the eukaryotic translation elongation factor 1 alpha (EEF1A1) internal control, with bars indicating fold change relative to cells grown in DMEM medium containing 2% Ultroser G. All predesigned TaqMan™ gene expression assays (Thermo Fisher Scientific, MA, US) are listed in Table 1.

**Table 1:** Gene target and TaqMan™ gene expression assay

Gene target	TaqMan™ gene expression assay
EEF1A1	Bt03223794_gl
PAX7	Hs00242962_ml
MYOD1	Bt03244740_ml
SDC4	Mm01179833_m1
MYF5	Bt03258928_ml
CCND1	Bt03235028_ml
IGF1R	Hs04220396_g1

### Statistical analysis

Each SC culture experiment was conducted with 2–4 technical well replicates and repeated at least twice with independent cell seedings (biological replicates) except the IncuCyte® data, which was performed once with technical triplicates. The isolated cells used in the experiment originated from one donor animal. Data are presented as mean ± SEM. Significant differences between treatments and the control sample were determined using one-way ANOVA or two-way ANOVA, as indicated in the figure legends. Differences were considered significant at *p* < 0.05. All statistical analyses were performed using GraphPad Prism version 9 (GraphPad Software, La Jolla, CA, USA).

## Supplementary information

Supplementary Information

## Data Availability

All data generated or analyzed during this study are included in this published article.
