# Supplementary material for: Cloning, expression and functional characterization of prepared bovine, salmon, and cod basic fibroblast growth factor-2
Source: NPJ Sci Food. 2025 Nov 21;9:248. doi: 10.1038/s41538-025-00609-2 (PMC12639020; doi:10.1038/s41538-025-00609-2)
Supplement: Supplementary file 1 — Supplementary Information [file 41538_2025_609_MOESM1_ESM.docx]

Supplementary data:

Cloning, Expression and Functional Characterization of Prepared Bovine, Salmon, and Cod Basic Fibroblast Growth Factor-2

S. Skrivergaard^1^, M. E. Pedersen^2#^, A. Holck^3^, A. Fagerlund^3^, L. Axelsson^3^, N. Solberg^2^, M. K. Rasmussen^1^, J. F. Young^1^, M. Therkildsen^1^, S.B. Rønning^2^

1 Department of Food Science, Aarhus University, Denmark

2 Nofima AS, Raw Materials and Optimization, Ås, Norway

3 Nofima AS, Food Safety and Quality, Ås, Norway

*** Correspondence:**

Corresponding Author:

Mona Elisabeth Pedersen, Osloveien 1, 1430 ÅS, Norway, tel +47 93405055, mona.pedersen@nofima.no

## Supplementary Table S1:

Codon-optimized synthesized sequences of the bovine, salmon, and cod *FGF2* genes.

| Gene | Codon-optimised synthesized gene |
| --- | --- |
| Bovine *FGF2* | **CATATG**AGAGGATCGCATCACCATCACCATCACGGATCTGGCTCTGGATCTGGTATCGAGGGAAGGATGCCCGCTCTACCAGAAGATGGAGGGTCAGGCGCATTTCCGCCAGGCCACTTTAAAGATCCGAAGCGCTTGTACTGCAAGAACGGCGGTTTTTTCCTGCGCATTCATCCGGACGGCCGTGTTGACGGCGTTCGTGAAAAATCGGACCCGCACATCAAGTTGCAACTGCAAGCGGAAGAGCGCGGTGTGGTGTCCATTAAGGGTGTGTGTGCGAATCGTTACCTGGCTATGAAAGAAGATGGCCGTTTGCTGGCGAGCAAGTGCGTCACCGATGAGTGCTTTTTCTTCGAGCGTCTTGAGTCTAACAACTACAATACCTATCGTAGCCGTAAATACAGCAGCTGGTATGTTGCGCTGAAACGCACCGGTCAGTATAAGCTGGGTCCGAAGACGGGTCCGGGTCAGAAAGCCATCCTGTTCCTCCCGATGAGCGCAAAATCCTAATA**GAATTC** |
| Salmon *FGF2* | **CATATG**AGAGGATCGCATCACCATCACCATCACGGATCTGGCTCTGGATCTGGTATCGAGGGAAGGATGCCCGCTACACCAGAAGATGGAGGGTCAGGCGGTTTTCCGCCAGGTAATTTTAAGGACCCGAAGCGCCTCTACTGCAAGAACGGTGGCTATTTCCTGCGTATTAACAGCAACGGCTCTGTGGACGGCATCCGCGAAAAAAACGATCCGCACATCAAGCTGCAACTGCAGGCGACCTCCGTGGGTGAGGTCGTGATCAAGGGTGTTTCGGCGAATCGTTACCTGGCTATGAATGGCGACGGCCGTTTGTTCGGCACCCGTCGCACGACCGATGAGTGTTACTTCATGGAACGTTTGGAGAGCAACAACTACAATACCTATCGTAGCCGTAAATACCCGGATATGTATGTTGCGCTGAAACGCACTGGTCAGTATAAAAGCGGTTCCAAGACCGGTCCGGGTCAAAAAGCAATTCTGTTTTTACCGATGTCTGCCCGTAGATAATA**GAATTC** |
| Cod *FGF2* | **CATATG**AGAGGATCGCATCACCATCACCATCACGGATCTGGCTCTGGATCTGGTATCGAGGGAAGGATGCCCGCTACACCAGATGACGGATCAGGGGGCTTTCCAGCGGCGAACTTTCGTGATCCGAAACGTCTGTACTGTAAAAACGGCGGTTTCTTTCTCCGTATTGCGAGCGACGGCCGCGTGGACGGGGTGCGTGAAAAGACCAATCCGCACATCCGCTTGCAACTGCAAGCGACGAGCGTTGGTGAAGTTGTCATCAAAGGTCTGGCCGCGAATCGTTACCTGGCTATGAACCGCGACGGCAGATTGTTCGGCGCACGTCGCGCAACCGATGAGTGCTACTTCCTTGAGCGCTTGGAGAGCAATAACTATAACACCTATCGTTCGAAAAAGTACCCGGAAATGTATGTTGCCCTGCAGCGTAGCGGTCAGTACAAGACTGGTACCAAAACCGGTCCGGGTCAGAAGGCGATTCTGTTCCTGCCGATGGCTTCTCGTTCCTAATA**GAATTC** |
| *Nde*I site and *Eco*RI site (used for cloning into the pET-30a(+) plasmid) are in bold. Start and stop codons are underlined. | |


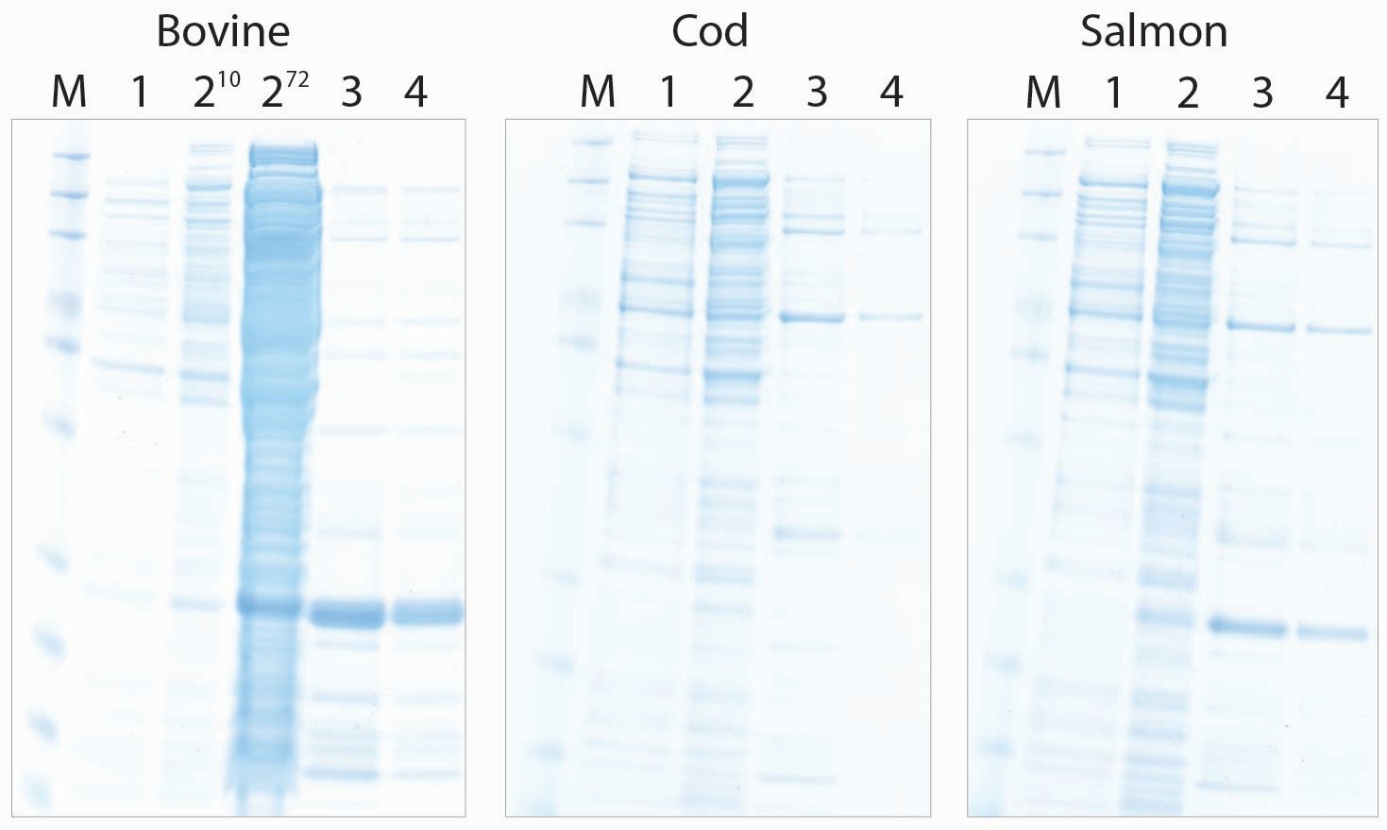


## Supplementary Figure S1

SDS-PAGE gels stained with Simply blue showing FGF-2 proteins before and after Ni-NTA purification. Lane 1: Uninduced sample taken immediately before addition of IPTG. Lane 2: Cleared lysate from induced cells expressing FGF-2, before purification. Superscripts indicate addition of 10 µg and 72 µg loaded protein, respectively. Lanes 3 and 4: FGF-2 after Ni-NTA purification, before and after desalting, respectively. M; molecular weight marker.


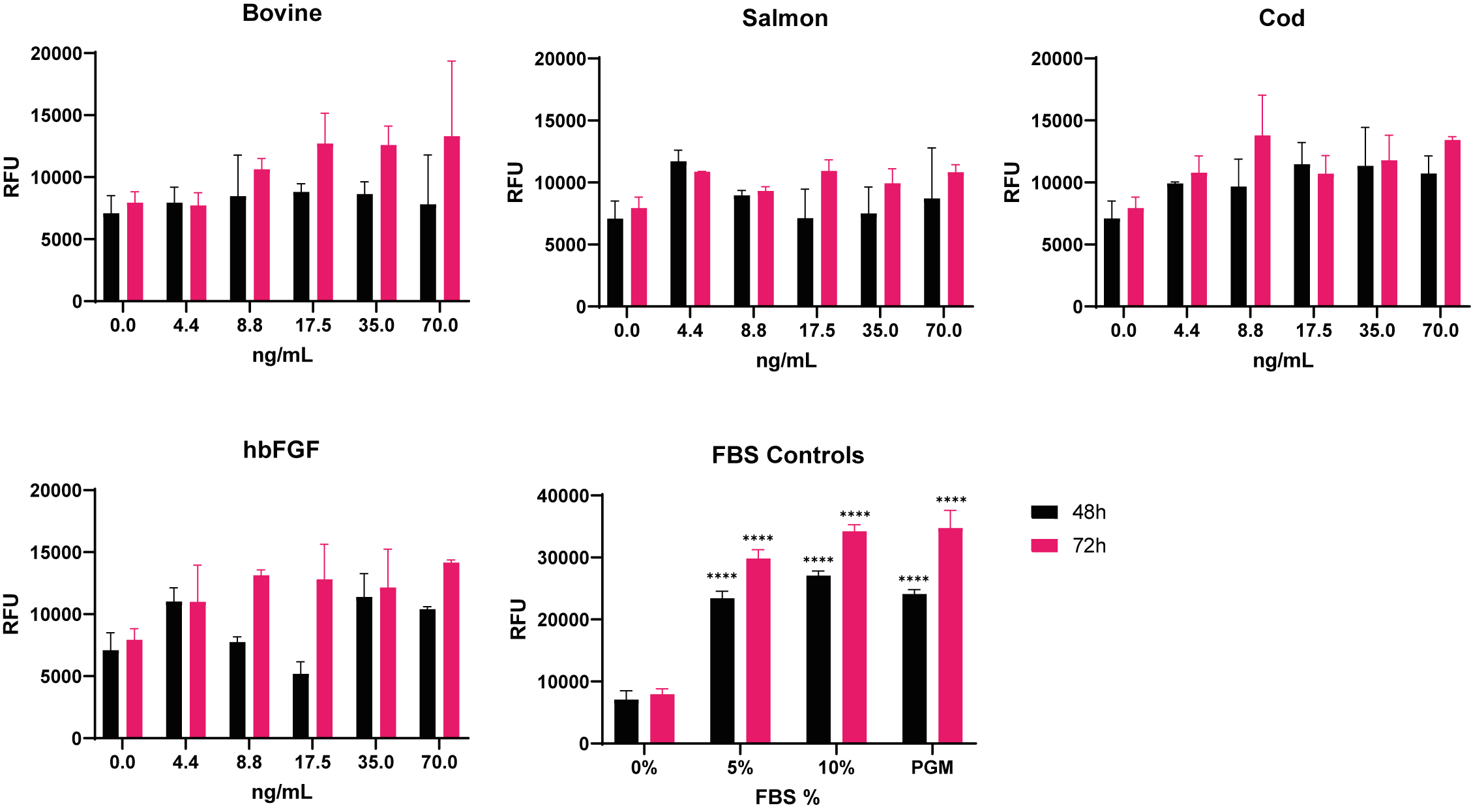


## Supplementary Figure S2

FGF-2 concentrations tested were between 0 and 70 ng/mL according to BioRad protein quantification. The FBS controls included basal medium with 0, 5 and 10% FBS, and PGM (proliferative growth medium), which was DMEM with 2% FBS and 2% Ultroser G. Cells were grown for 48 and 72 h. The response was measured as relative fluorescence units (RFU). Two-way ANOVA with Dunnett’s multiple comparisons to 48 hours in which p < 0.05 (*), p < 0.01 (**), p < 0.001 (***), p < 0.0001 (****). FGF-2 samples n = 2, FBS control samples n = 4. Error bars are ±SEM.


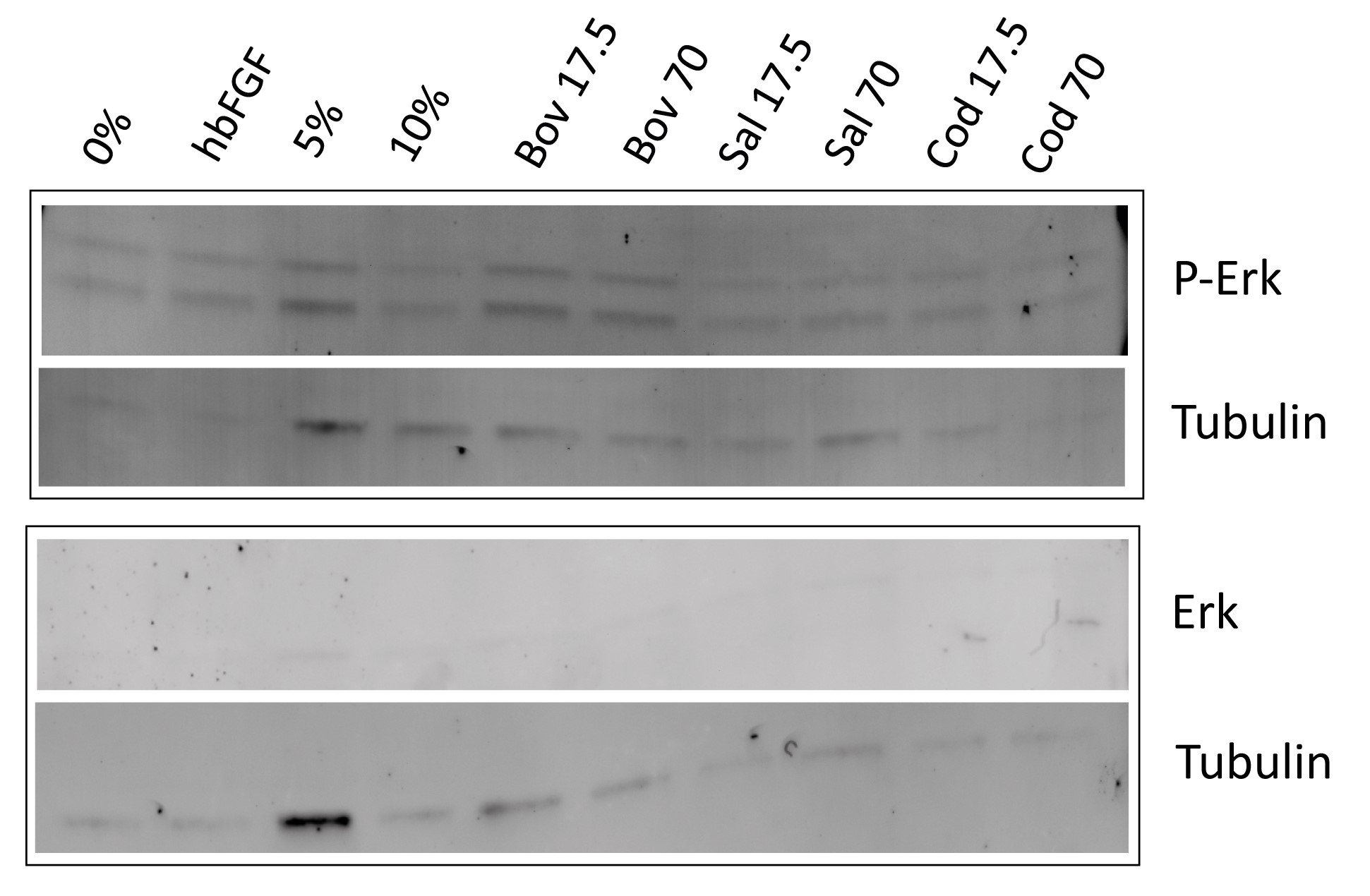


## Supplementary Figure S3:

WB of cells incubated with 17.5 ng/mL hbFGF, 5% or 10% FBS, or the three in-house FGF-2 variants at 17.5 ng/mL and 70 ng/mL for 20h in basal medium. Blots grouped in black boxes are from the same membrane.

## Supplementary Figure S4:

CyQuant DNA quantification of cells incubated with Mek or p38 inhibitors in different concentrations in basal medium (0%), basal medium with hbFGF at 17.5 ng/mL or in PGM for 72h. 2-way ANOVA with Dunnett’s multiple comparisons to the control sample in each treatment group in which p < 0.05 (*), p < 0.01 (**), p < 0.001 (***), p < 0.0001 (****). Control samples n = 8, while inhibitor samples n = 4. Error bars are ±SEM.


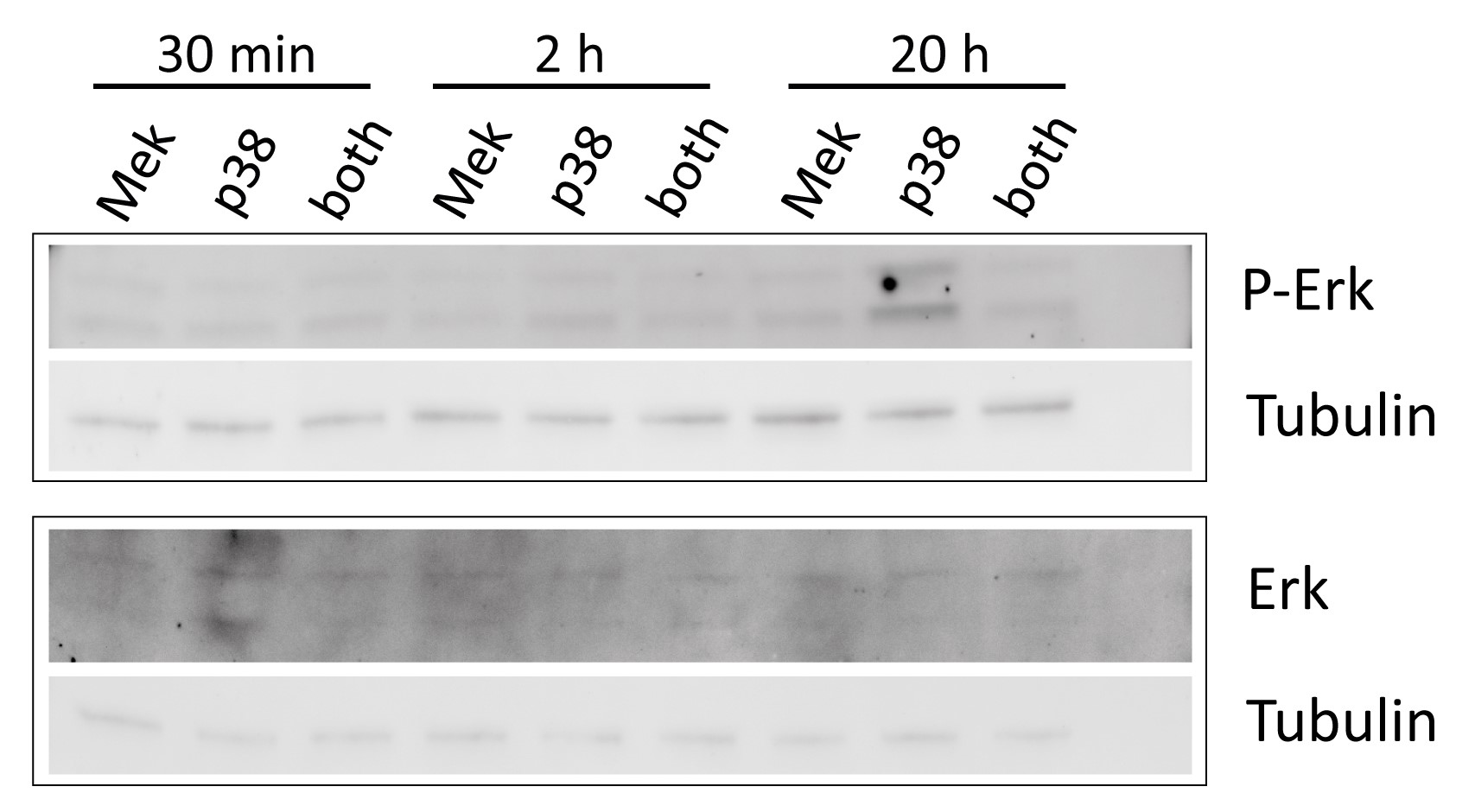


## Supplementary Figure S5:

WB of cells incubated in 5% FBS with either Mek or p38 or both inhibitors present for 30 minutes, 2 or 20 hours. Blots grouped in black boxes are from the same membrane.

## Supplementary Figure S6:

IncuCyte proliferation assays use FGF-2 in combination with fetuin and ITS for salmon and cod FGF-2, with concentrations based on either the BioRad method (total protein quantification) or quantified according to the band on a gel (gel-adjusted quantification) using 70 ng/mL and 2 ng/mL concentrations.

##
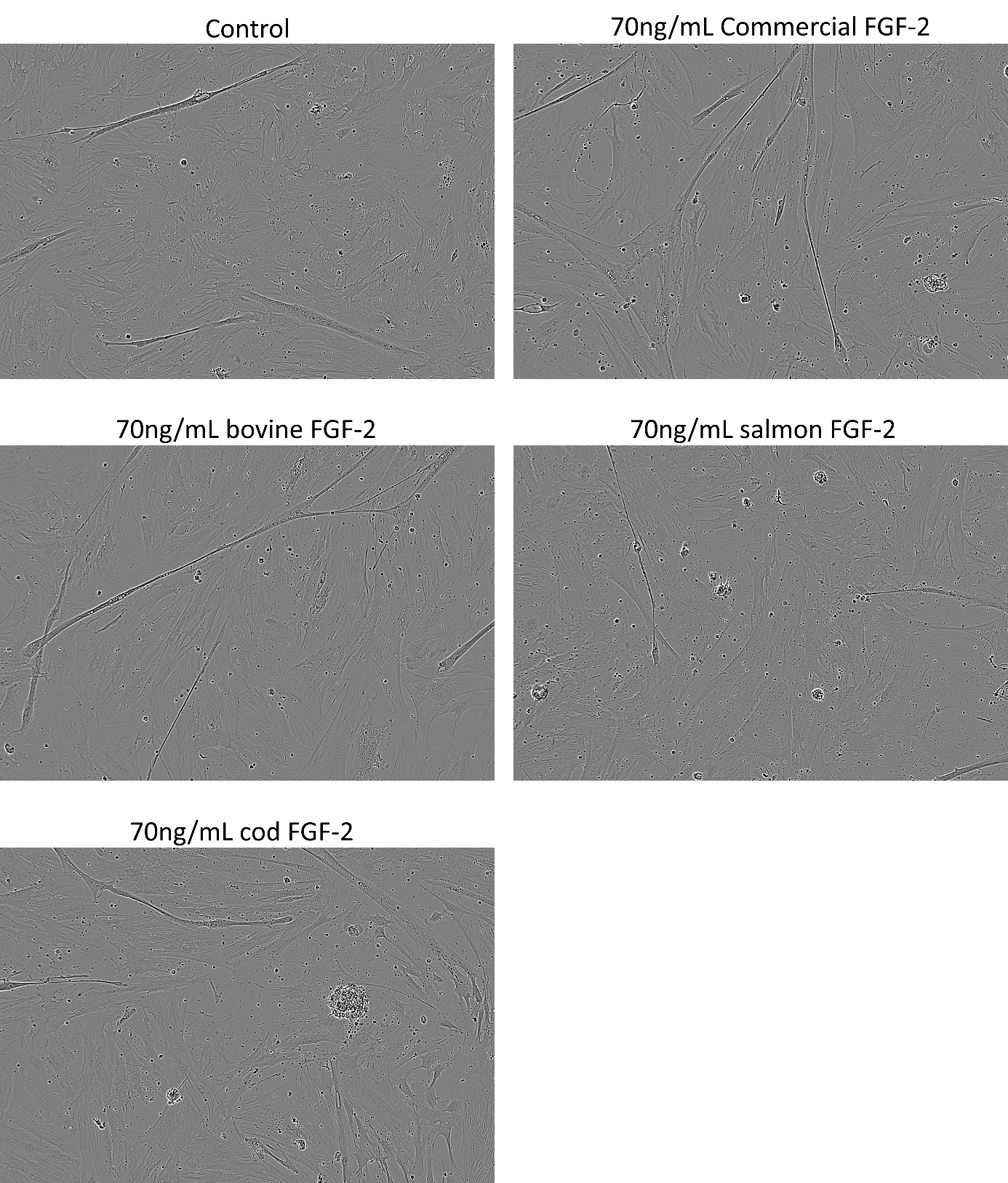


## Supplementary Figure S7:

Primary bovine myoblast cells are able to differentiate after cultivation in both commercial and homemade FGF-2. Cells were grown to confluency for 6 days before they were cultivated in differentiation media for 5 days. Control cells were grown in growth media without FGF-2. Pictures are acquired by the IncuCyte S3 Live Cell analysis system.


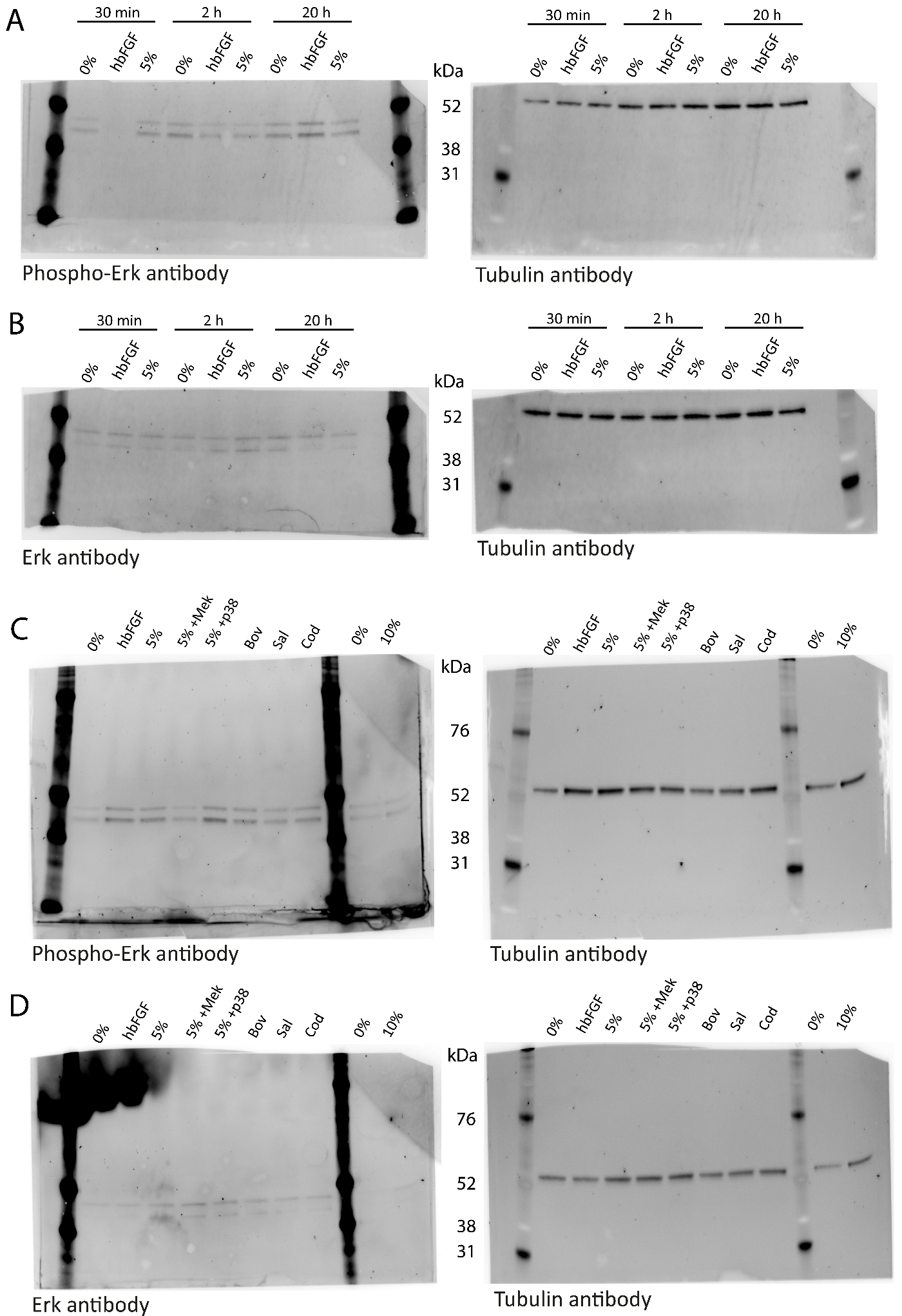


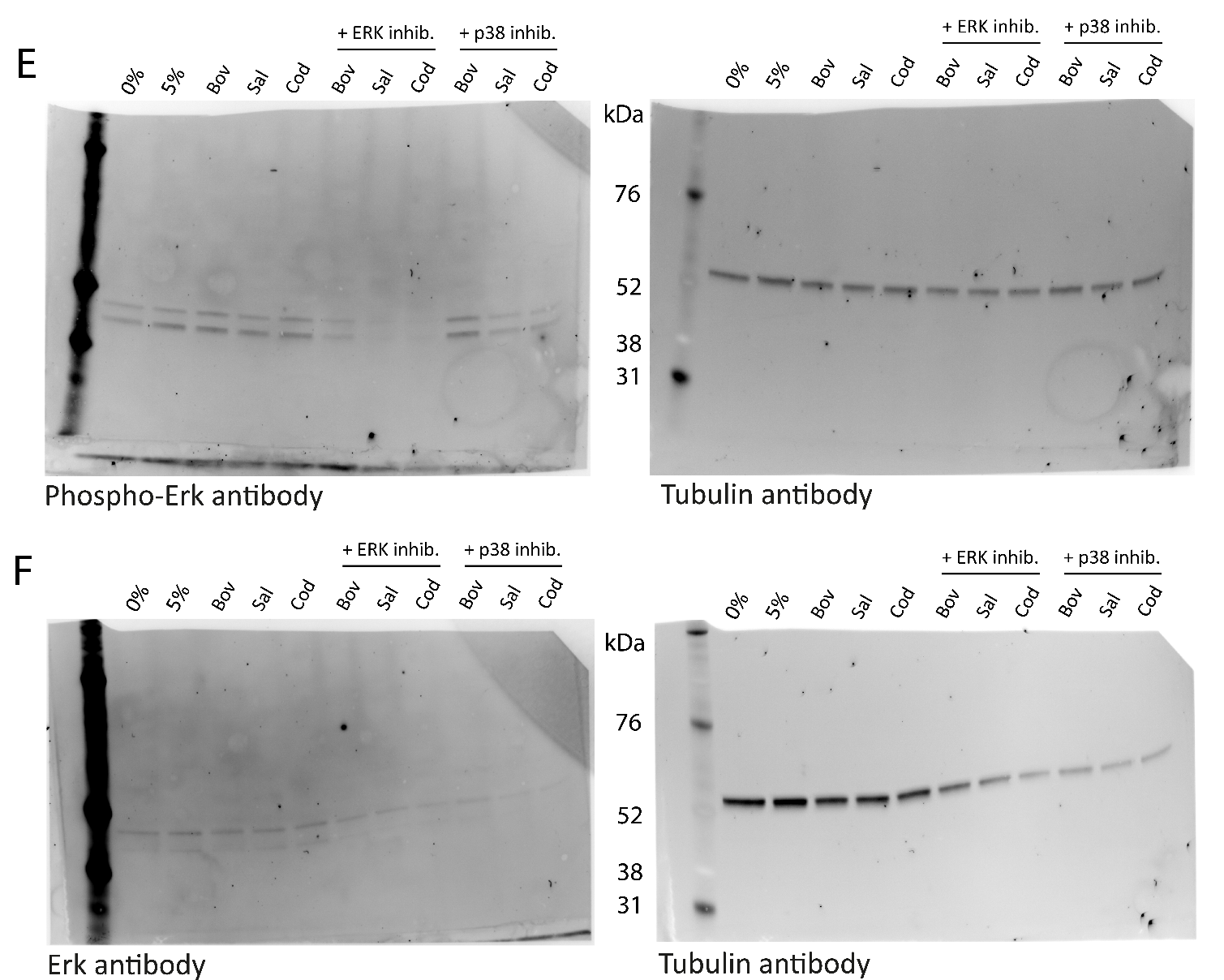


##
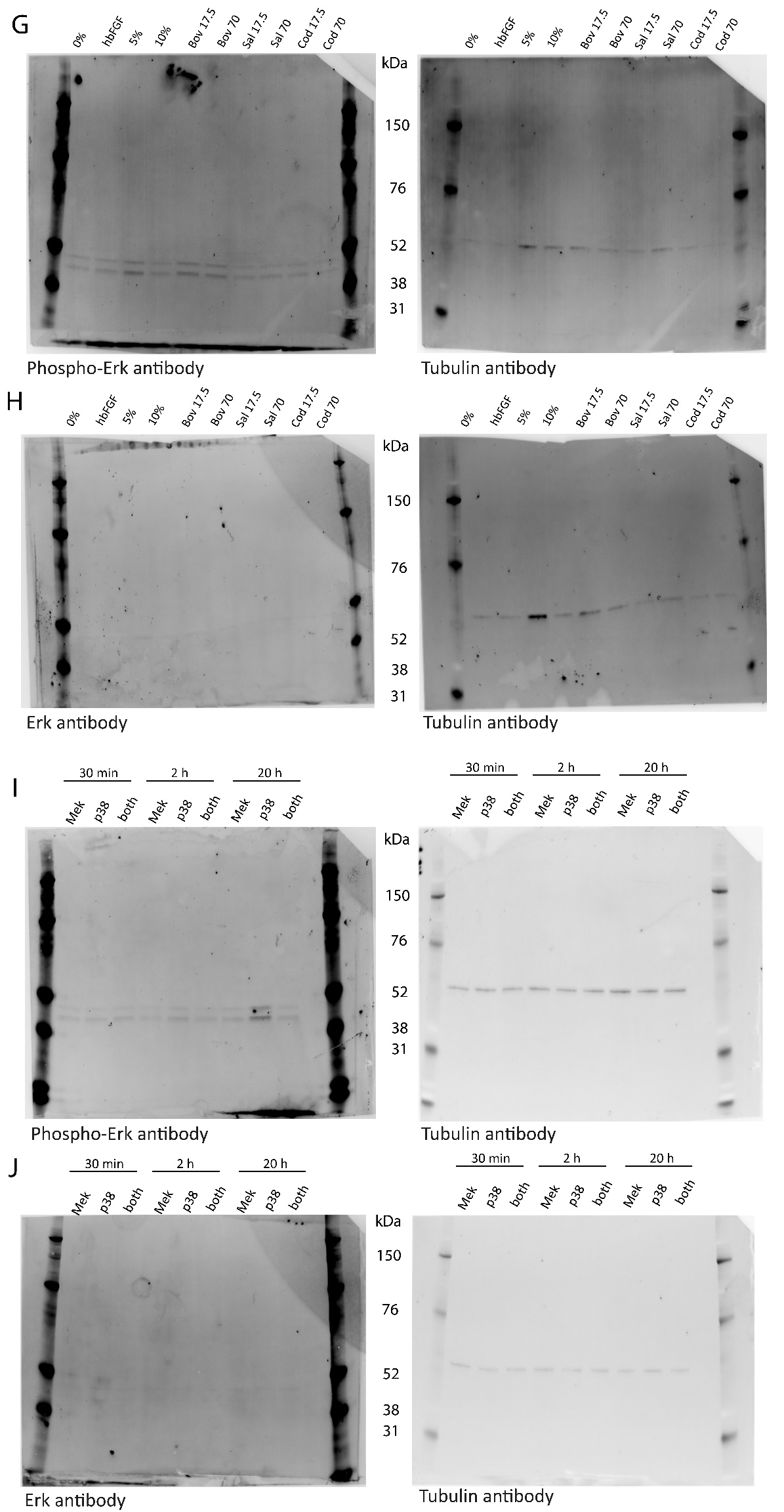
Supplementary Figure S8:

Original uncropped Western blots with size (in kDa) indicated. **A+B** are blots related to Fig. 3B. **C+D** are blots related to Fig. 3C and 4A. **E+F** are blots related to Fig. 4A. **G+H** are blots related to Fig. S2. **I+J** are blots related to Fig. S4.

##
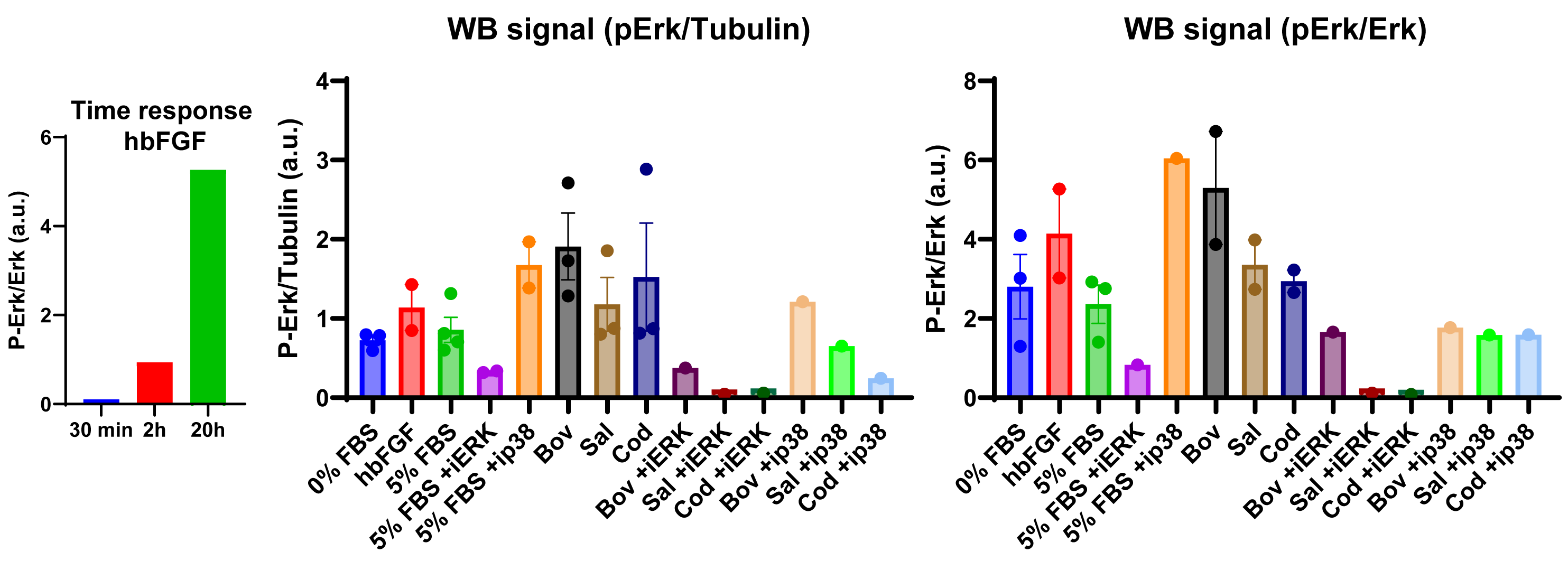


## Supplementary Figure S9:

Western blots were quantified using ImageJ in arbitrary units (a.u.) using the area under the curve with the Gel Plot analysis tool. P-Erk and Erk were quantified as the sum of the two isoform bands. P-Erk/Tubulin signal numbers. P-Erk/Erk signal numbers are based on the P-Erk relative to total Erk both being normalized against Tubulin. Low replicate numbers (n = 1-4) prevented proper statistical analysis. Mean values are shown with error bars ±SEM.
